# Association between cancer risk assessment tool use and GP consultation duration: an observational study

**DOI:** 10.3399/BJGP.2024.0135

**Published:** 2025-04-08

**Authors:** Emily Fletcher, John L Campbell, Emma Pitchforth, Luke Mounce, Willie Hamilton, Gary Abel

**Affiliations:** National Institute for Health and Care Research senior journal editor, Global Health Research and executive editor, Sexual and Reproductive Health Matters;; National Institute for Health and Care Research senior journal editor, Global Health Research and executive editor, Sexual and Reproductive Health Matters;; National Institute for Health and Care Research senior journal editor, Global Health Research and executive editor, Sexual and Reproductive Health Matters;; University of Exeter Medical School, Exeter, Devon.; University of Exeter Medical School, Exeter, Devon.; University of Exeter Medical School, Exeter, Devon.

**Keywords:** consultations, diagnosis, electronic clinical decision support, general practice, risk, workload

## Abstract

**Background:**

England is short of GPs, and GP consultation rates, consultation duration, and workload are increasing. Electronic clinical decision support tools assist decision making for screening, diagnosis, and risk management. Cancer detection is one area in which tools are designed to support GPs, with some electronic risk assessment tools (eRATs) estimating the risk of current cancer based on symptoms.

**Aim:**

To explore any association between the impact of eRATs and GP workload and workflow during consultations.

**Design and setting:**

Observational sub-study.

**Method:**

Thirteen practices in England participating in a cluster randomised controlled trial of eRATs were recruited to the study. Using mixed-effects regression models, the average duration of consulting sessions and individual consultations in which eRATs were, or were not, activated were compared.

**Results:**

There was no evidence that consulting sessions in which an eRAT was activated were, on average, longer than sessions in which an eRAT had not been activated. However, after adjusting for a range of session and consultation characteristics, individual consultations involving an eRAT were longer, on average, by 3.96 minutes (95% confidence interval = 3.45 to 4.47; *P*<0.001) when compared with consultations with no eRAT.

**Conclusion:**

There was no evidence to suggest that eRATs should not be used to support GPs in early cancer diagnosis from a workload perspective. Activation of eRATs was not associated with increased workload across a consulting session, despite a small increase in time observed in individual consultations involving eRATs. Ultimately, therefore, it should be definitive findings regarding the clinical effectiveness of eRATs, not the related workload/workflow implications, that determine whether the use of eRATs should be rolled out more widely.

## Introduction

GPs manage an increasing and complex workload.^1^ For the last decade at least, England’s GP workforce has been in crisis: a third of GPs have reported their intention to quit within 5 years.^2^^–^^8^ Workload increases and a depleted GP workforce are likely to impact on GPs’ ability to manage competing demands in a 10-minute consultation.^9^^,^^10^

Electronic clinical decision support (eCDS) tools assist clinicians in detecting and managing various health conditions.^11^^–^^15^ Cancer electronic risk assessment tools (eRATs) are a particular type of eCDS developed to support GPs. Late diagnosis of cancer and its initial detection in primary care are key reasons why the UK has lower cancer survival rates than other countries.^16^ eRATs contain algorithms that access patient data in an electronic medical record (EMR); this is similar to other cancer tools, such as C the Signs (https://www.cthesigns.com) and QCancer.^11^^,^^17^^–^^20^ It is not known what impact eCDS tools have on workload and workflow during GP consultations, although clinicians commonly expect that consultation workload will increase or workflow will be disrupted.^21^^–^^23^ Longer consultations have been shown to be associated with GPs’ stress levels^24^^–^^27^ and, although they do not capture tasks conducted outside of consultations,^28^^–^^30^ understanding whether eCDS tools are associated with impacts on consultation duration, workflow, and the duration of subsequent consultations in the same session may help to facilitate their implementation.

This study aimed to assess the impact of eRATs on GP workload and workflow by measuring consultation duration. The objective was to determine the average difference in duration of consulting sessions and of individual consultations in which an eRAT alert was, or was not, activated. An additional objective was to compare the average duration of all consultations in a session that followed a consultation in which an eRAT was activated with that of consultations in sessions in which eRATs had not been activated.

**Table:** How this fits in

Pressure and workloads are increasing in general practice, and a large number of experienced GPs are leaving the workforce. Particular efforts are being made to help GPs spot the early signs of cancer and many electronic clinical decision support systems have been designed to raise GPs’ awareness of individual patients in whom cancer symptoms could be considered. Previously, it was not known what the implications of using such potentially interruptive tools during a consultation would be, in terms of GP workload measured by time. This study sheds light on the fact that, although an individual consultation may be slightly lengthened, there is no indication that GP workload increases across a whole consulting session in terms of time.

## Method

### Design and sampling

An observational sub-study was conducted within the Electronic Risk Assessment for Cancer (ERICA) trial. ERICA aims to explore the clinical effectiveness of GPs using eRATs embedded in their IT systems, compared with usual care.^31^^–^^32^ The sample size for the observational study was based on the number of consultations expected to occur over a 2-week period, using GP workforce data from NHS Digital.^33^^,^^34^

Power calculations were based on previous estimates of the standard deviation of consulting session and individual consultation durations — namely, 20 minutes and 4 minutes, respectively.^35^^–^^37^ Data on 2 weeks of consultations from 15 practices were expected to provide >80% power to detect a difference of 10 minutes in session duration and 2 minutes in consultation duration between sessions and consultations in which eRAT alerts were/were not activated, even if eRAT alerts were to affect only 2.5% of sessions. Minimally important differences of 2 minutes to 5 minutes for individual consultation length, and 10 minutes for consulting session length were agreed in advance within the research team based on team experience and expertise.

The authors sought to recruit up to 15 ERICA practices in England (see Supplementary Information S1) where the eRAT software had successfully been installed and was being actively used by GPs, as identified from routine usage reports from that software. Practices received £100 in acknowledgement of their time.

### Outcomes

The two primary outcomes were:

- duration (in minutes) of whole consulting sessions; and
- duration (in minutes) of individual consultations.

A consulting session was defined as a half-day period of pre-booked or same-day consultations.^38^ A consultation was defined as starting when the patient’s EMR was opened by a GP, preceding a face-to-face or telephone interaction with a patient, and ending on EMR closure (captured in the SystmOne records system).^39^ Home visits and consultations with other health professionals who had no access to eRATs during the ERICA trial were excluded.

The secondary outcome was the duration (in minutes) of consultations that took place immediately after a consultation in which an eRAT alert had been activated within the same consulting session.

### Data collection

Data on consultations were extracted from practice IT systems for a minimum of 2 weeks in each practice during 2022 and 2023, using a search method from previous work.^39^ Data on GPs’ usage of eRATs were extracted from the eRATs software. Instances of eRAT alerts were cross-referenced with the consultations data; a full description of data items and the method of matching eRAT alerts to sessions and consultations is presented in Supplementary Information S2. Five practices piloted the data extraction procedures.

### Analysis

Data were analysed in Stata (version 18). The primary analyses of the duration of consulting sessions and individual consultations took the form of mixed-effects linear regression. At session level, random intercepts accounted for clustering of sessions within GPs, and for GPs clustering within practices; at consultation level, intercepts also included consultations clustering within sessions. The regression analyses adjusted for time of day*,* day of week*,* consultation mode*,* proportion of telephone versus face-to-face consultations in the session, and total number of consultations in the session. The consultation regression analysis also adjusted for the order/position of a consultation within a session. Bootstrapping was undertaken, and sensitivity analyses explored the implications of uncertainty and assumptions made in the primary analyses (Supplementary Boxes S1–S3). There was no control group of practices for this study, so within-group comparisons were made.

The secondary analysis also used mixed-effects linear regression to compare the duration of all consultations occurring after a consultation involving an eRAT (within the same session) with a matched group of consultations undertaken by the same GP 1 week later in a session in which no eRAT alert was activated. A similar set of random intercepts were applied as for the primary consultations analysis, but the random intercept accounting for consultation clustering within sessions was replaced with one for a ‘matched’ pair of sessions. The precise methods for matching consultations with those occurring 1 week later are described in Supplementary Information S2.

## Results

**Figure 1.**
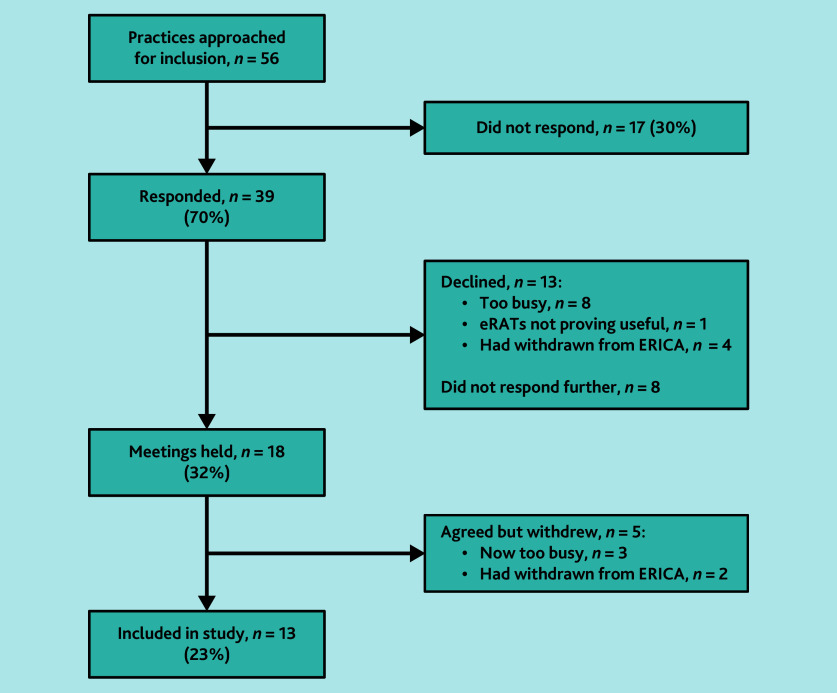
Study recruitment process. eRAT = electronic risk assessment tool. ERICA = Electronic Risk Assessment for Cancer.

Thirteen practices were recruited (Figure 1), with a total of 60 GPs across these practices all having used eRATs since August 2021. Data were collected across the practices between September 2022 and August 2023.

Table 1 summarises practice characteristics. Most practices (54%) had a ‘medium’ urgent suspected cancer referral ratio, 38% had a ‘high’ referral ratio, and just one practice (8%) had a ‘low’ referral ratio. Mean list size was 12 775 patients, with an average headcount of 11 GPs. The mean Index of Multiple Deprivation (IMD) decile score was 3.14, indicating that practices were in areas of lesser deprivation.

**Table 1.** Practice characteristics, *n* = 13

**Characteristic**	**Value**
**Age–sex standardised USC referral ratio, *n* (%)^a^**	
Low	1 (8)
Medium	7 (54)
High	5 (38)

**List size, *n* (%)^b^**	
Small: <3500	0 (0)
Medium: 3500–8000	4 (31)
Large: >8000	9 (69)

**List size, mean (range)^b^**	12 775 (4027–44 920)

**IMD 2019 score, mean (range)^c^**	3.14 (1–6)

**GP headcount, mean (range)^d^**	11.36 (3–40)

a *The number of USC referrals by GPs, standardised according to the practice’s list size, and to the age and sex distributions of patients on the list. The ratio was computed by the ratio of the observed number of referrals to the expected number of referrals (expected number calculated from age- and sex-specific list-size data for each practice, and from national age- and sex-specific ratios of urgent referral), with ‘low’, ‘medium’, and ‘high’ defined according to national tertiles.^34^*

b *The number of patients registered at a GP practice, according to official statistics from NHS Digital.^40^*

c *IMD 2019 overall score (1–10) in national deciles to reflect the relative deprivation of practices’ catchment areas (higher score indicates greater deprivation).^41^*

d *Practice list size and GP count for each practice, according to NHS Business Services Authority data.^42^ IMD = Index of Multiple Deprivation. USC = urgent suspected cancer.*

The total number of eRATs activated in a practice during data collection (Table 2, column 7) was larger in all cases than the number of eRATs successfully matched to a session (Table 2, column 8) or to an individual consultation (Table 2, column 9). A major reason for this was eRAT alerts being activated on a day when a GP was not consulting but, perhaps, had opened a patient’s EMR for another task. In addition, multiple cancer eRAT alerts could be activated within a consultation, and eRAT alerts were occasionally activated between consultations: the authors could not attribute these to either the prior or subsequent consultation and hence were excluded from the main analyses.

**Table 2.** Practice descriptive statistics

**Practice**	**Practice consultation data**					**Practice eRATs data**		

	**GPs using eRATs, *n* (%)^a^**	**Consulting sessions, *n***	**Mean session duration, minutes**	**Mean consultations per session, *n***	**Mean consultation duration, minutes**	**Total eRATs, *n***	**eRAT sessions, *n* (%)^b^**	**eRAT consultations, *n* (%)^c^**
**1**	13 (68)	687	212.94	14.04	13.84	1344	96 (14)	175 (2)
**2**	5 (50)	502	228.56	12.00	16.86	252	41 (8)	66 (1)
**3**	6 (43)	508	282.72	14.90	15.53	647	86 (17)	192 (3)
**4**	1 (20)	594	207.51	14.73	11.01	141	18 (3)	22 (0.1)
**5**	8 (42)	945	229.79	12.00	15.30	705	92 (10)	161 (1)
**6**	3 (60)	388	259.02	15.10	12.29	552	86 (22)	149 (3)
**7**	3 (33)	247	277.51	17.66	12.25	69	12 (5)	16 (0.4)
**8**	5 (38)	289	172.50	9.43	15.05	1237	107 (37)	165 (6)
**9**	2 (50)	317	216.27	11.70	16.45	298	18 (6)	44 (1)
**10**	1 (20)	332	198.36	14.64	12.35	29	6 (2)	10 (0.2)
**11**	4 (100)	339	208.34	12.20	13.19	554	45 (13)	80 (2)
**12**	7 (50)	790	149.39	11.48	12.33	234	8 (1)	9 (0.1)
**13**	2 (50)	213	152.43	9.85	14.11	20	2 (1)	2 (0.1)

a *Percentage of GPs in the practice.*

b *Percentage of consulting sessions in which at least one eRAT alert was activated.*

c *Percentage of individual consultations in which at least one eRAT alert was activated. eRAT = electronic risk assessment tool.*

### Primary analysis

#### Consulting sessions

Sessions in which an eRAT alert was activated were 11.85 minutes longer, on average (95% confidence interval [CI] = 3.95 to 19.76; *P*<0.05), than those in which no eRAT alert was activated (unadjusted) (Table 3). After adjusting for time of day, day of week, number of consultations in the session, and the proportion of telephone consultations in the session, this observed difference reduced to 5.92 minutes (95% CI = −1.52 to 13.35; *P* = 0.119), which was no longer a statistically significant difference.

Afternoon/evening sessions were 36.01 minutes shorter, on average, than morning sessions (95% CI = −40.27 to −31.76; *P* <0.001) (Table 3). In addition, sessions with larger proportions of telephone consultations were shorter, on average, than those with a smaller proportion; as an example, sessions with 25%–50% telephone consultations were shorter by 10.05 minutes (95% CI = −19.35 to −0.75), and sessions with >75% telephone consultations were shorter by 18.51 minutes (95% CI = −26.88 to −10.13); *P*<0.001. The day of the week was not associated with a difference in session duration (*P* = 0.191).

**Table 3.** Results of regression analyses examining the duration of consulting sessions

**Category**	**Unadjusted**		**Adjusted**	

	**Consulting session length, minutes (95% CI)**	***P*-value**	**Consulting session length, minutes (95% CI)**	***P*-value**
**eRAT activation**				
No eRAT activated in session	Reference	0.003	Reference	0.119
eRAT activated in session	11.85 (3.95 to 19.76)		5.92 (−1.52 to 13.35)	

**Time of day**				
Morning	Reference	<0.001	Reference	<0.001
Afternoon/evening	−57.48 (−61.77 to −53.19)		−36.01 (−40.27 to −31.76)	

**Day of week**				
Monday	Reference	0.382	Reference	0.191
Tuesday	1.52 (−4.89 to 7.93)		6.38 (−0.37 to 13.13)	
Wednesday	4.90 (−1.78 to 7.93)		2.43 (−3.80 to 8.66)	
Thursday	0.50 (−6.00 to 7.00)		4.60 (−0.93 to 10.13)	
Friday	−1.80 (−8.65 to 5.04)		4.80 (−0.47 to 10.08)	

**Total number of consultations in the session (difference per additional consultation)**	10.33 (9.82 to 10.84)	<0.001	9.14 (−19.35 to −0.75)	<0.001

**Proportion of telephone consultations in the session, %**				
<25%	Reference	0.007	Reference	<0.001
25–50%	−11.37 (−22.19 to −0.56)		−10.05 (−19.35 to −0.75)	
50–75%	0.54 (−10.05 to 11.12)		−9.87 (−18.19 to −1.56)	
>75%	−0.29 (−13.63 to 7.05)		−18.51 (−26.88 to −10.13)	

a *Unless otherwise stated. eRAT = electronic risk assessment tool.*

Session duration varied, on average, by 31.25 minutes between practices (95% CI = 28.42 to 33.49), and by 35.13 minutes between GPs (95% CI = 33.05 to 37.35); that is, neither clustering had an overwhelmingly larger influence, but practice had a slightly larger influence than GP.

#### Individual consultations

Consultations involving an eRAT alert were longer, on average, by 5.85 minutes (95% CI = 5.25 to 6.45; *P*<0.001) compared with consultations in which no eRAT was activated (unadjusted) (Table 4).

**Table 4.** Results of regression analyses examining the duration of individual consultations

**Category**	**Unadjusted**		**Adjusted**	

	**Consultation length, minutes (95% CI)**	***P*-value**	**Consultation length, minutes (95% CI)**	***P*-value**
**eRAT activation**				
no eRAT activated	Reference	<0.001	Reference	<0.001
eRAT activated	5.85 (5.25 to 6.45)		3.96 (3.45 to 4.47)	

**Time of day**				
Morning	Reference	0.972	Reference	<0.001
Afternoon/evening	0.00 (−0.15 to 0.16)		−0.57 (−0.74 to −0.40)	

**Day of week**				
Monday	Reference	<0.001	Reference	0.106
Tuesday	0.46 (0.21 to 0.71)		−0.25 (0.05 to 0.46)	
Wednesday	0.16 (−0.07 to 0.39)		0.09 (−0.10 to 0.29)	
Thursday	0.54 (0.30 to 0.77)		0.15 (−0.06 to 0.35)	
Friday	0.36 (0.11 to 0.60)		0.06 (−0.16 to 0.28)	

**Total number of consultations in the session (difference per additional consultation)**	−0.23 (−0.25 to −0.22)	<0.001	−0.21 (−0.23 to −0.20)	<0.001

**Proportion of telephone consultations, %**				
<25%	Reference	<0.001	Reference	<0.001
25–50%	0.18 (−0.17 to 0.54)		1.80 (1.46 to 2.15)	
50–75%	−0.94 (−1.28 to −0.61)		2.56 (2.26 to 2.86)	
>75%	−2.65 (−2.97 to −2.34)		2.62 (2.33 to 2.90)	

**Consultation mode**				
Face-to-face	Reference	<0.001	Reference	<0.001
Telephone	−6.33 (17.25 to 17.53)		−6.46 (−6.63 to −6.30)	

**Order/position of consultation in the session**	4.59 (4.02 to 5.15)	<0.001	1.28 (16.76 to 17.48)	0.028

*eRAT = electronic risk assessment tool.*

After adjusting for time of day, day of week, total number of consultations in the session, proportion of telephone consultations in the session, consultation mode (telephone or face-to-face), and order/position of the consultation within the session, this observed difference reduced to, on average, 3.96 minutes (95% CI = 3.45 to 4.47; *P*<0.001) longer. In general, afternoon/evening consultations were shorter than morning consultations by, on average, 0.57 minutes (or 34.2 seconds) (95% CI = −0.74 to −0.40; *P*<0.001). Telephone consultations were shorter, on average, by 6.46 minutes (95% CI = −6.63 to −6.30; *P*<0.001) than face-to-face consultations. In sessions with larger proportions of telephone versus face-to-face consultations — that is, >50% telephone — consultations conducted by telephone were shorter on average than those face-to-face by up to 2.62 minutes (95% CI = 2.33 to 2.90; *P*<0.001).

There was no evidence that day of week was associated with a difference in consultation duration (*P*=0.106).

Consultation duration varied, on average, by 1.52 minutes (95% CI = 1.44 to 2.51) between practices, by 2.60 minutes (95% CI = 2.45 to 2.74) between GPs, and by 0.58 minutes (or 34.8 seconds) between sessions (95% CI = 0.56 to 0.61). The biggest influence on duration was, therefore, variation between GPs, with session variation having the smallest influence.

None of the sensitivity analyses affected the results of the primary analyses (Supplementary Tables S1 and S2).

### Secondary outcomes

#### Subsequent consultations

There was no evidence that consultations that followed one in which an eRAT had been activated differed in average duration when compared with those that occurred at the same timepoint 1 week later, with the same GP, in sessions involving no eRATs: difference = 0.21 minutes (or 12.6 seconds) (95% CI = −0.59 to 1.01; *P* = 0.606) (Table 5). After adjusting for the other factors in the model, this result reduced to 0.12 minutes (or 7.2 seconds) (95% CI = −0.59 to 0.83; *P* = 0.735). Variation seemed to be mostly influenced by practice, with consultations varying, on average, by 2.53 minutes between practices (95% CI = 1.83 to 3.51); variation was, on average, 1.84 minutes (95% CI = 0.95 to 3.54) between GPs, and consultations were not noticeably influenced by session.

**Table 5.** Duration of consultations occurring after an eRAT was activated

**Category**	**Unadjusted**		**Adjusted**	

	**Consultation length, minutes (95% CI)**	***P*-value**	**Consultation length, minutes (95% CI)**	***P*-value**
**eRAT activation**				
eRAT not activated in the session	Reference	0.606	Reference	0.735
eRAT activated in the session	0.21 (−0.59 to 1.01)		0.12 (−0.59 to 0.83)	

**Total number of consultations in the session**	−0.17 (−0.28 to −0.05)		−0.10 (−0.20 to −0.00)	0.047

**Proportion of telephone consultations in the session, %**				
<25	Reference	<0.001	Reference	0.169
25–50	−0.98 (−4.02 to 2.07)		1.22 (−1.23 to 3.67)	
50–75	−1.53 (−4.74 to 1.67)		2.67 (−0.20 to 4.74)	
>75	−3.52 (−6.71 to −0.33)		2.32 (−0.03 to 4.67)	

**Consultation mode**				
Face-to-face	Reference	<0.001	Reference	<0.001
Telephone	−7.36 (−8.23 to −6.48)		−7.48 (−8.48 to −6.49)	

**Order position of consultation in the session**	−0.77 (−2.53 to 0.99)	0.389	0.38 (−1.38 to 2.15)	0.670

*eRAT = electronic risk assessment tool.*

## Discussion

### Summary

This study explored the impact of eRAT alerts on the duration of consulting sessions and individual consultations as a measure of GP workload and workflow. There was no evidence that sessions during which an eRAT alert had been activated were longer than those with no eRAT, after adjusting for consultation factors and accounting for potential confounding by practice or GP. However, individual consultations involving an eRAT alert were longer, by 3.96 minutes on average, than consultations that did not involve an eRAT. Thus, although there was a noticeable difference to consultation duration, this did not translate into any impact on overall workload over the course of a whole session. These findings were not sensitive to making alterations to the key underlying assumptions (relating to factors such as individual consultations appearing to last <1 minute or >60 minutes, consulting sessions of <60 minutes duration or with fewer than 6 consultations, or the precise timing of eRAT alert in relation to the timing of the consultation or consulting session, Supplementary Boxes S1 and S2) applied to the data in the primary analysis at session or individual consultation level.

Analysis of the duration of consultations that took place in a session after an eRAT alert had been activated showed no evidence that they were longer or shorter, on average, than those occurring in a session with no eRATs.

### Strengths and limitations

This work used a robust method to extract, match, and interpret routine consultation duration data from a UK primary care system and usage data from an eCDS tool for analysis, which could be extended to research into other types of eCDS tools. The method was not burdensome; no access to the content of the EMR was required, and nor was there any extraction from the EMR of sensitive information. Necessary data was extracted from the appointment system, not from the patient’s EMR. Capturing duration data by video-or audio-recording consultations, or clinicians self-reporting timings, all have practical and ethical challenges. Recruitment took place after the COVID-19 pandemic; as such, practices were used to using remote video-conferencing for meetings, and no travel costs or additional time were required for the study.

There is a limitation relating to the GP population that participated in this study; the sample cannot be considered to be representative of all English GPs, and those who took part were from participating practices that had a research background. The authors are, therefore, unable to comment on the wider generalisability of their findings to UK general practice; the results are also not necessarily transferable to GPs who are unfamiliar with, or unmotivated to use, the tools employed. The SystmOne EMR records system was the only system in which the eRATs had been successfully implemented at the time of recruitment to the sub-study. The tools became operational within another EMR system (EMIS) later, but this occurred at too late a stage for these practices to be included, which would have increased the generalisability of the findings. It was also not possible to collect GPs’ age, gender, ethnicity, or years of experience, which would have been useful to examine.^37^

An ideal comparator for consultations involving an eRAT would have been those in control practices from the ERICA trial; however, the authors could not involve control practices due to practical and ethical difficulties in identifying consultations that would have activated an eRAT alert based on symptom codes in the EMR. The comparator consultations in intervention practices may have, therefore, not reflected consultations taking place in control-arm practices.

### Comparison with existing literature

Clinicians expect eCDS tools to add consultation time, and this is a commonly cited barrier to the implementation of such tools.^43^ Sittig *et al*^44^ reported that physicians were reluctant to use an eCDS if they were worried that it would lead to more tests; >84% of participants reported being *‘more than 20 minutes behind’* either some, most, or all of the time, and were less likely to respond to alerts when running late. Much of the existing evidence is qualitative or mixed methods in nature, with fewer studies having directly focused on measuring consultation duration.^43^

Kostopolou *et al*^45^ found that eCDS use in simulated consultations did not increase duration or test ordering. Further, Porat *et al*^46^ (same study) found that 13 GPs who had expressed concerns about time took longer to consult when using the eCDS (average duration 15.45 minutes) than in their baseline session (average 13.53 minutes), but this was not the case for the GP sample as a whole.

Other research indicates that consultations towards the end of a consulting session tend to be shorter than earlier consultations.^26^ This may reflect GPs feeling pressure to see patients in a timely manner or their having a different case mix of patients, including ‘extras’ at the end of a session with more acute illnesses. In the findings presented here, an eRAT may have extended a consultation, but either there was no noticeable impact on the duration of the consultations that followed or GPs attempted to keep to time; it is not possible to know which of these (or other) interpretations are true.

Although eRATs did not add to workload, in terms of time, to a whole consulting session, there may have been workload implications within consultations that were not measurable by time, such as a future appointment being booked for a patient to discuss cancer risk. In addition, different forms of workload (for example, emotional or cognitive) may be incurred, which also do not affect consultation duration. Dual process theory describes two levels of reasoning:^47^ theoretically, when they are consulting, GPs mostly use type 1 thinking — which involves automated processes during busy sessions;^48^ type 2 processes require more cognitive analytical effort, particularly when something unexpected occurs (for example, an eRAT alert), which is incongruent with the patient’s presenting complaint.

### Implications for research

The context for this research was the use of eRATs for cancer risk estimation.^11^^,^^49^^,^^50^ The methods used by the authors of the study presented here, however, could be applied to investigate the workload implications of other similar tools that present risk information — such as those alerting GPs to a patient’s risk of cardiovascular disease, as highlighted by previous research,^51^^,^^52^ or other cancer risk assessment tools, such as QCancer and C the Signs.

A main highlighted weakness of this research was that comparisons were not made with consultations in which an eRAT would have been activated if the software were present. This could be addressed, with future research embedding inactive tools in practices (subject to ethical considerations) or through an examination of routine electronic health records data. However, given the absence, or modest magnitude, of the duration differences found, it is not clear that such studies are warranted.

Reports highlight that heavy workload contributes to GP fatigue, as well as affecting patient safety and practice team functioning.^53^^,^^54^ The Royal College of General Practitioners has argued that 10-minute consultations should be extended to 15 minutes,^55^ particularly as England has shorter consultations than other countries; the Australian average duration has remained stable at 15 minutes, and the average duration in the US has steadily increased to >20 minutes.^56^ The observation that consultations involving an eRAT alert were longer, on average supports the idea that longer consultations may be necessary when a cancer diagnosis is a possibility, or where an alert has been raised.

The authors did not capture associations between eRATs and GP workload outside of a consultation, such as during the days/weeks following an eRAT alert, when referral/investigation activities may occur. The findings may, therefore, underestimate the impact of eRATs on workload, suggesting that, at a minimum, they are associated with an increased length of the index consultation. However, the results indicate that there are few reasons, from a workload perspective, why eRATs should not be used in general practice to assist with early cancer diagnosis.

## Data

Data generated or analysed during this study have been included in this article. Further details available from the authors on reasonable request.
